# Scales and Dermal Skeletal Histology of an Early Bony Fish *Psarolepis romeri* and Their Bearing on the Evolution of Rhombic Scales and Hard Tissues

**DOI:** 10.1371/journal.pone.0061485

**Published:** 2013-04-09

**Authors:** Qingming Qu, Min Zhu, Wei Wang

**Affiliations:** 1 Subdepartment of Evolution and Development, Department of Organismal Biology, Evolutionary Biology Centre, Uppsala University, Uppsala, Sweden; 2 Key Laboratory of Evolutionary Systematics of Vertebrates of Chinese Academy of Sciences, Institute of Vertebrate Paleontology and Paleoanthropology, Chinese Academy of Sciences, Beijing, China; Ecole Normale Supérieure de Lyon, France

## Abstract

Recent discoveries of early bony fishes from the Silurian and earliest Devonian of South China (e.g. *Psarolepis*, *Achoania*, *Meemannia*, *Styloichthys* and *Guiyu*) have been crucial in understanding the origin and early diversification of the osteichthyans (bony fishes and tetrapods). All these early fishes, except *Guiyu*, have their dermal skeletal surface punctured by relatively large pore openings. However, among these early fishes little is known about scale morphology and dermal skeletal histology. Here we report new data about the scales and dermal skeletal histology of *Psarolepis romeri*, a taxon with important implications for studying the phylogeny of early gnathostomes and early osteichthyans. Seven subtypes of rhombic scales with similar histological composition and surface sculpture are referred to *Psarolepis romeri*. They are generally thick and show a faint antero-dorsal process and a broad peg-and-socket structure. In contrast to previously reported rhombic scales of osteichthyans, these scales bear a neck between crown and base as in acanthodian scales. Histologically, the crown is composed of several generations of odontodes and an irregular canal system connecting cylindrical pore cavities. Younger odontodes are deposited on older ones both superpositionally and areally. The bony tissues forming the keel of the scale are shown to be lamellar bone with plywood-like structure, whereas the other parts of the base are composed of pseudo-lamellar bone with parallel collagen fibers. The unique tissue combination in the keel (i.e., extrinsic Sharpey's fibers orthogonal to the intrinsic orthogonal sets of collagen fibers) has rarely been reported in the keel of other rhombic scales. The new data provide insights into the early evolution of rhombic (ganoid and cosmoid) scales in osteichthyans, and add to our knowledge of hard tissues of early vertebrates.

## Introduction

**Figure 1:**
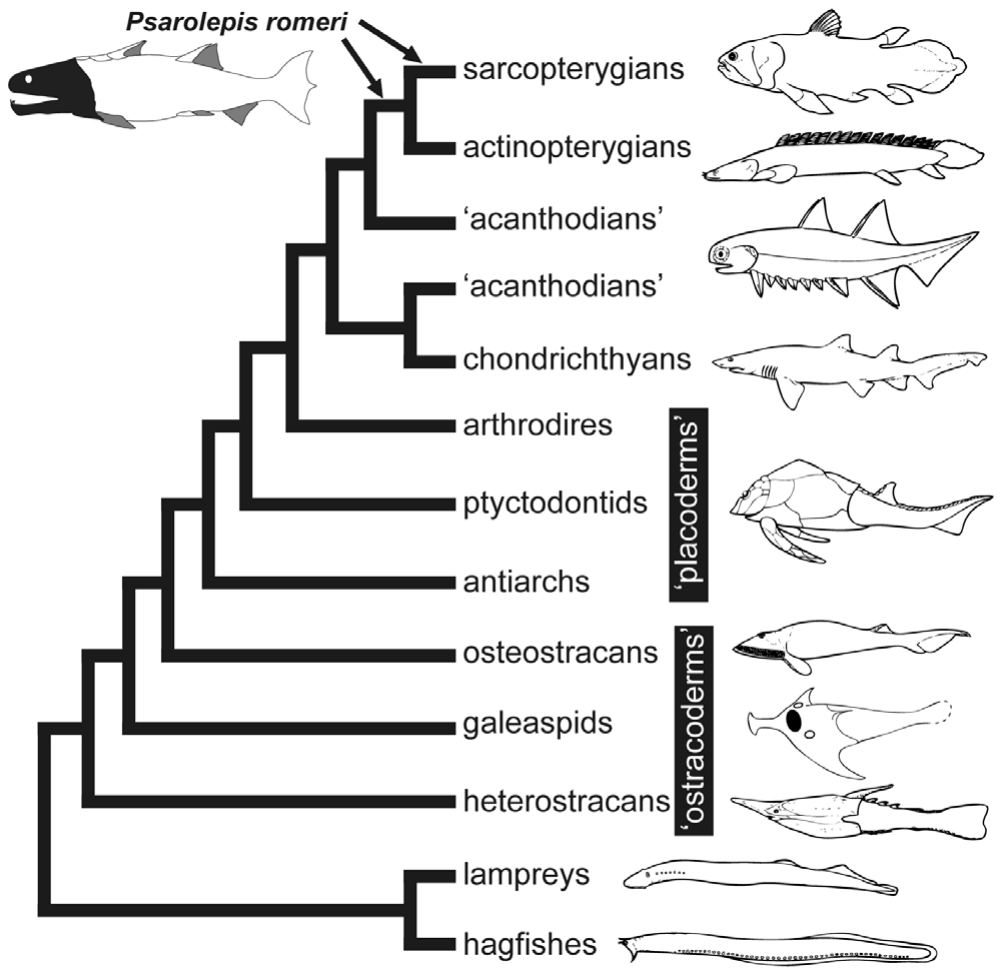
The phylogenetic framework showing the alternative positions of *Psarolepis romeri*. Based on references [3], [6], [7], [8]. Icons of representative fishes after reference [9].

*Psarolepis romeri*, from the Pridoli (Silurian) and Lochkovian (Devonian) of South China [1], [2], [3] and the late Silurian of Vietnam [4], is one of the earliest known sarcopterygians (lobe-finned fishes and tetrapods). Initially referred to crown sarcopterygians (Dipnomorpha sensu Ahlberg [5])[2], *Psarolepis* was soon assigned to either the osteichthyan or sarcopterygian stem based on cladistic analysis [3] (Figure 1, based on references [3], [6], [7], [8], [9]). Later phylogenetic studies except that of Zhu and Schultze [10] have generally resolved *Psarolepis* as a stem sarcopterygian (e.g. [7], [11]). The morphological reconstruction of *Psarolepis* was based on disarticulated remains [3], and has been corroborated by its close relative *Guiyu*, the oldest articulated osteichthyan from the Ludlow (Silurian), South China [11], [12]. The dermal skeleton of *Guiyu* lacks cosmine, a unique sarcopterygian tissue complex [13], [14], [15], [16]; *Psarolepis* thus represents the oldest known sarcopterygian with cosmine-like tissue complex, with the potential to contribute to the understanding of the origin of cosmine, as well as the dermal skeleton of early osteichthyans.

Wang [17] mentioned the abundant occurrence of the surface-pore-bearing scales from the Xitun Formation (Lochkovian) of Yunnan, which corresponds to the high diversity of early sarcopterygians in this stratum [3], [11], but only the trunk scales of *Styloichthys* have been briefly described [18], [19]. This work on the scales of *Psarolepis* represents the starting point for detailed study of squamation of the osteichthyans discovered in the Xitun Formation.

The dermal skeletal histology of *Psarolepis* and *Styloichthys* was illustrated briefly by Zhu et al. [20], for the purpose of the comparison with the histology of the coeval *Meemannia*. Zhu et al. [21] gave a more thorough description of the histology of *Meemannia* and provided detailed information for further comparative studies. The present work provides additional description of the dermal skull histology of *Psarolepis* and reveals histological differences, such as the shape of pore cavities and diverse hard tissue resorption conditions, among the early osteichthyans from the Xitun Formation. The dermal skull histology will also be compared with the scale histology, thus serving as complementary evidence for our proposed taxonomic assignment of the disarticulated scales.

## Materials and Methods

This study is based on ground sections of a parietal shield (IVPP V17756) and isolated scales of *Psarolepis* from the Early Devonian bone beds of the Xitun Formation in Qujing, East Yunnan, China. All the scales in this study were extracted by treatment with dilute acetic acid (10%) from greenish-grey argillaceous limestone of the Xitun Formation.

All material to be sectioned was first embedded in light-curing embedding resin Technovit 7200. Ground sections were made through three planes (antero-posterior vertical, dorso-ventral vertical and horizontal) for each subtype of scales. When making the ground sections, the sample was first glued to a glass slide using the same resin for embedding. Then the other surface was ground until the preset surface of the specimens was exposed, and this surface was glued to another glass slide. A diamond wafer-cutting blade mounted on the EXAKT-300CL band system was used to cut the second glass slide (with 100–200 µm of the specimen in the resin) off the whole sample. Finally the second glass was ground to about 20–30 µm manually using grit sizes ranging from P1200 to P4000. In this way, 2–3 ground sections out of one scale can be made for those scales larger than 1 mm in depth. But for dorso-ventral vertical ground sections, only one section could be made from one scale. All the ground sections were examined and photographed using transmitted and polarized light microscopy (Leica Photomicroscopy with Nomarski Differential Interference Contrast (DIC) at Department of Organismal Biology, Uppsala University, Sweden).

Two scales were sectioned in antero-posterior vertical direction after embedding. Sectioning surfaces were then etched for 40–60 seconds using 1% phosphoric acid. After that, they were washed and dried, and coated with gold before SEM study using Hitachi S-3700N at the Key Laboratory of Evolutionary Systematics of Vertebrates, Institute of Vertebrate Paleontology and Paleoanthropology (IVPP). All material is housed in IVPP, China.

## Results

### (a) Assignment of the scales

Recent work using acid treatment of rock samples from the Xitun Formation has recovered large numbers of scales bearing large pores on their surface, in addition to acanthodian, thelodont, and placoderm scales. Previously described microfossils from the Xitun Formation include acanthodian scales and jaw fragments, thelodont scales, and putative chondrichthyan scales and teeth [17], [22]. So far, seven surface-pore-bearing forms have been described from the Xitun Formation based on macrofossil material (mostly cranial and/or isolated postcranial elements): *Youngolepis*, *Diabolepis*, *Psarolepis*, *Achoania*, *Styloichthys*, *Meemannia* and an onychodont-like form [2], [3], [18], [20], [23], [24], [25], [26].

Among the surface-pore-bearing rhombic scales recovered in the process, one type of scale manifests similar surface ornamentation characterized by relatively large pores (resembling the surface sculpture in *Psarolepis*, *Achoania*, *Styloichthys* and *Meemannia*) while revealing differences in scale morphology (e.g. depth to length ratio and peg-and-socket structure). Thus this type of scale is further classified into 7 subtypes according to their morphological differences (see ‘**Scale Morphology**’). Careful examination of scale subtypes shows that they all exhibit similar histological composition, suggesting that they belong to a single taxon. Although seven surface-pore-bearing forms have been reported from the same beds, we can use the method of exclusion to assign this special type of scale, based on comparison of histology and surface sculpture.

Histological information exists for five out of the seven surface-pore-bearing forms from the Xitun Formation, based on the lower jaw of *Youngolepis* [27], dermal skull of *Diabolepis* [27], dermal skull of *Meemannia* [20], [21], dermal shoulder girdle of *Styloichthys* [20] and dermal skull of *Psarolepis* [20].

**Figure 2:**
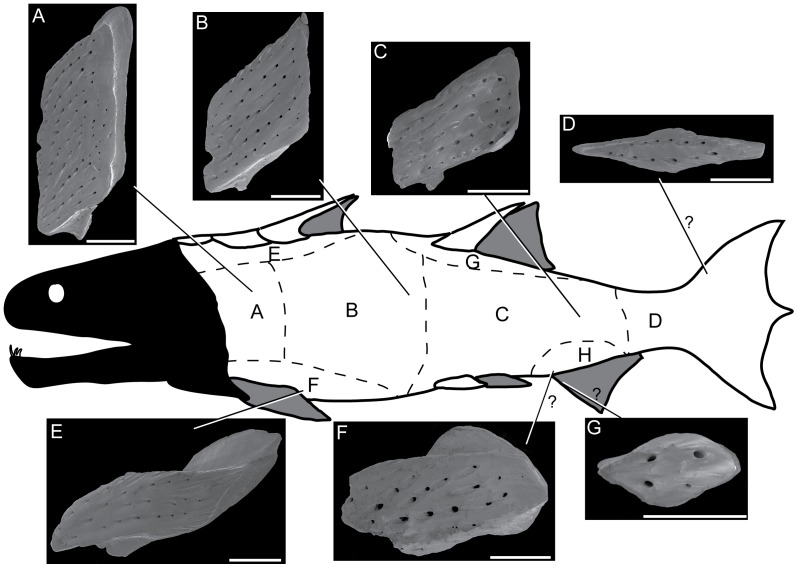
SEM photos of scales that probably constitute the squamation of *Psarolepis romeri*. **A.** IVPP V17913.6, subtype 1. **B.** IVPP V17913.7, subtype 2. **C.** IVPP V17913.8, subtype 3. **D.** IVPP V17913.9, subtype 4. **E.** IVPP V17913.10, subtype 5. **F.** IVPP V17913.11, subtype 6, note that this image has been mirrored in order to match the orientation of other scales. **G.** IVPP V17913.12, subtype 7. All scales in crown view and anterior to the right. Hypothetical outline of *Psarolepis* is adopted from *Guiyu* [11] with the squamation scheme from Esin [33]. Scales in A, B, C, E, and F come from the right side of the fish. Scale bar = 0.5 mm.

**Figure 9:**
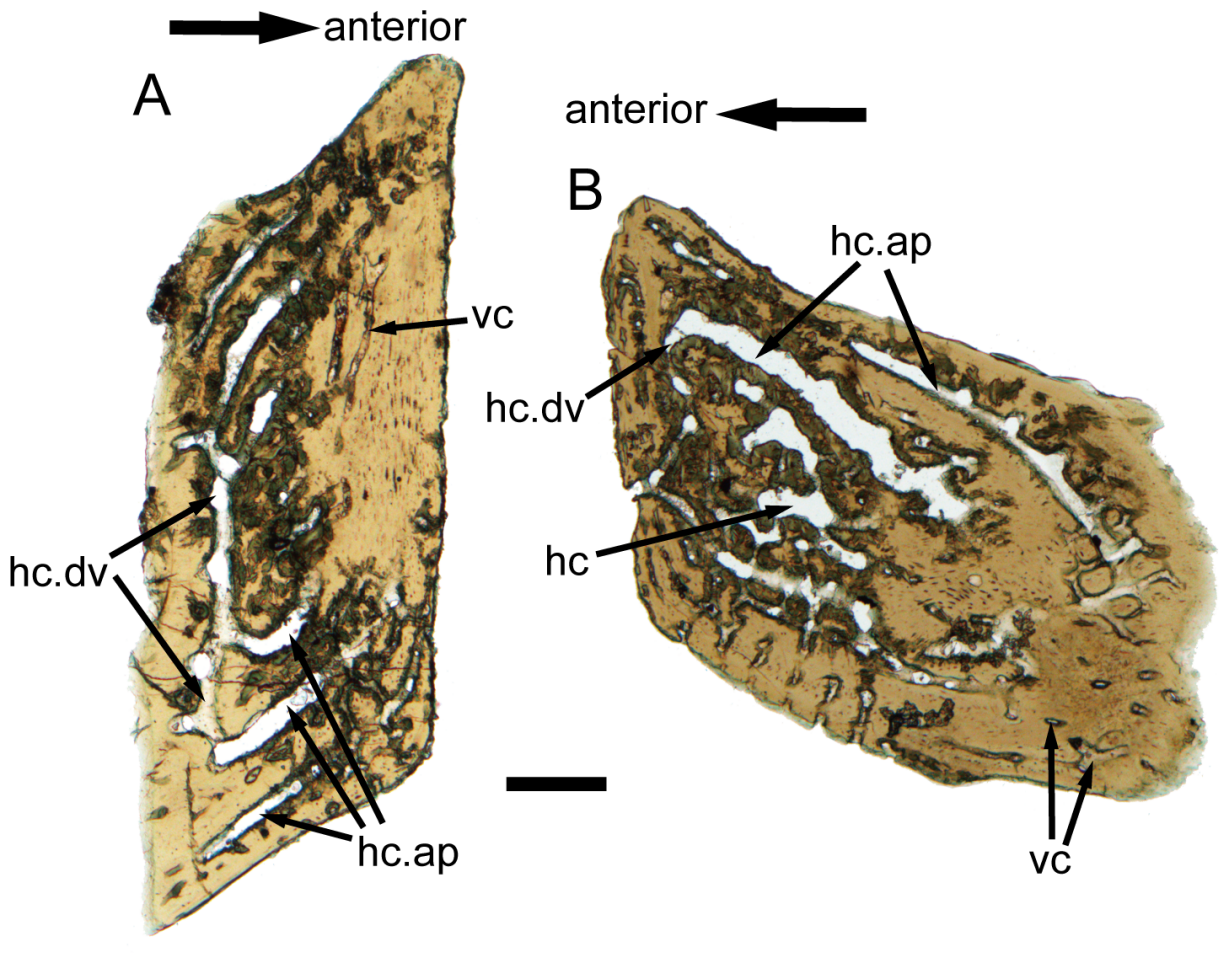
Horizontal ground sections of scales (subtypes 1 and 3) of *Psarolepis romeri*. **A.** IVPP V17757.29, subtype 1, horizontal ground section, light microscope photo, note that the dorso-ventrally and antero-posteriorly oriented horizontal canals join to form a horizontal canal network, where the pore cavities ascend from. **B.** IVPP V17757.30, subtype 3, horizontal ground section, light microscope photo. Scale bar = 200 µm. hc, horizontal canal; hc.dv, dorso-ventrally oriented horizontal canal; hc.ap, antero-posteriorly oriented horizontal canal; vc, vascular canal.

The referred scales in this study differ histologically from *Youngolepis* and *Diabolepis*, in addition to the obvious difference in size and distribution pattern of surface pores. While these scales reveal multiple layers of enamel plus dentine (superimposed odontodes), *Youngolepis* and *Diabolepis* ([27]: figs. 2 and 9) have one single layer of enamel, similar to ‘true cosmine’ [28] in other sarcopterygians such as *Porolepis*, *Osteolepis* and *Dipterus* [13]. In addition, *Youngolepis* and *Diabolepis* have flask-shaped pore cavities ([27]: figs. 2 and 9) instead of cylindrical pore cavities.

The referred scales differ histologically from *Styloichthys* because their buried odontodes always lie above the horizontal canal network, and never reach the underlying bony tissues (see ‘**Scale Histology**’). In *Styloichthys*, as in other rhipidistian sarcopterygians such as *Porolepis* [13] and *Uranolophus* [29], [30], the odontodes are deeply buried in the underlying bony tissues ([20]: fig. 2e).

The referred scales differ histologically from *Meemannia* because this taxon has flask-shaped pore cavities and superimposed odontodes (though lying above the horizontal canal network) show only superpositional growth pattern rather than both superpositional and areal growth patterns [20], [21].

On the other hand, the referred scales bear typical histological features found in known materials of *Psarolepis* (e.g. dermal skull) in the cylindrical pore cavities and the co-existence of both superpositional and areal growth patterns. These histological similarities are confirmed by new ground sections of dermal skeleton from a parietal shield of *Psarolepis* in this work (see the description of histology below).

Thus far, no histological information is available for *Achoania* (only based on one anterior portion of the skull [25], with five lower jaw specimens [26] and one shoulder girdle [31] assigned to the genus) and the onychodont-like form (only based on an incomplete lower jaw [26]), consequently no histological comparison can be made with these two poorly represented forms. However, as the referred scales in this study make up about 50% of all surface-pore-bearing scales in the entire sample, it is reasonable to assign the referred scales to *Psarolepis*, which is abundantly represented among macrofossils, rather than to the poorly represented *Achoania* or the onychodont-like form.

While this assignment based on similarities in histology and dermal surface sculpture, and the relative abundance of specimens, must remain tentative pending the discovery of articulated *Psarolepis* specimen with squamation, previous assignment of isolated *Psarolepis* materials (shoulder girdles, cheek plates, median fin spines, and most recently pelvic girdles) has received indirect corroboration from articulated *Guiyu* specimens in terms of the reconstructed body form and the restored position of isolated elements [11], [32].

Although we cannot exclude the possibility that *Achoania* may have scales similar to the scales here referred to *Psarolepis*, the overall significance of the scales referred to *Psarolepis* as described below would not be affected, as *Achoania* and *Psarolepis* are closely related to each other in most phylogenetic analyses (e.g. [11]). While recognizing the tentative nature of the assignment of these isolated scales, we believe that the morphological and histological details revealed by these scales will add to our understanding of *Psarolepis*, contribute to the ongoing discussion of the phylogenetic position of *Psarolepis* (either as a stem sarcopterygian or a stem osteichthyan), and bear on the study of ganoid and cosmoid scales in early bony fishes (see ‘**Discussion**’).

### (b) Scale morphology

Seven subtypes (subtypes 1–7, Figure 2) are recognized among the referred scales. Of 80 scales used for ground sections, 24 scales can be allocated to subtype 1, 17 scales to subtype 2, 19 scales to subtype 3, 11 scales to subtype 4, 3 scales to subtype 5, 1 scale to subtype 6, and 5 scales to subtype 7. The 7 subtypes are tentatively assigned to different regions of the body (Figure 2), based on the squamation scheme of Esin [33], which has been applied to the squamation of some osteichthyans with only disarticulated specimens in certain circumstances (e.g. [34], [35]). Studies on articulated specimens also support that this scheme is generally valid for early osteichthyans with rhombic scales (e.g. [11], [36]). Although the referred scales in this study are morphologically distinct, especially for being thick with a distinctive neck that has not been observed in other types of rhombic scales, our squamation model is inferential and can only be tested by articulated specimens of *Psarolepis*.

Below we first describe the shared features of the referred scales, and then the specific features for each subtype.

**Figure 3:**
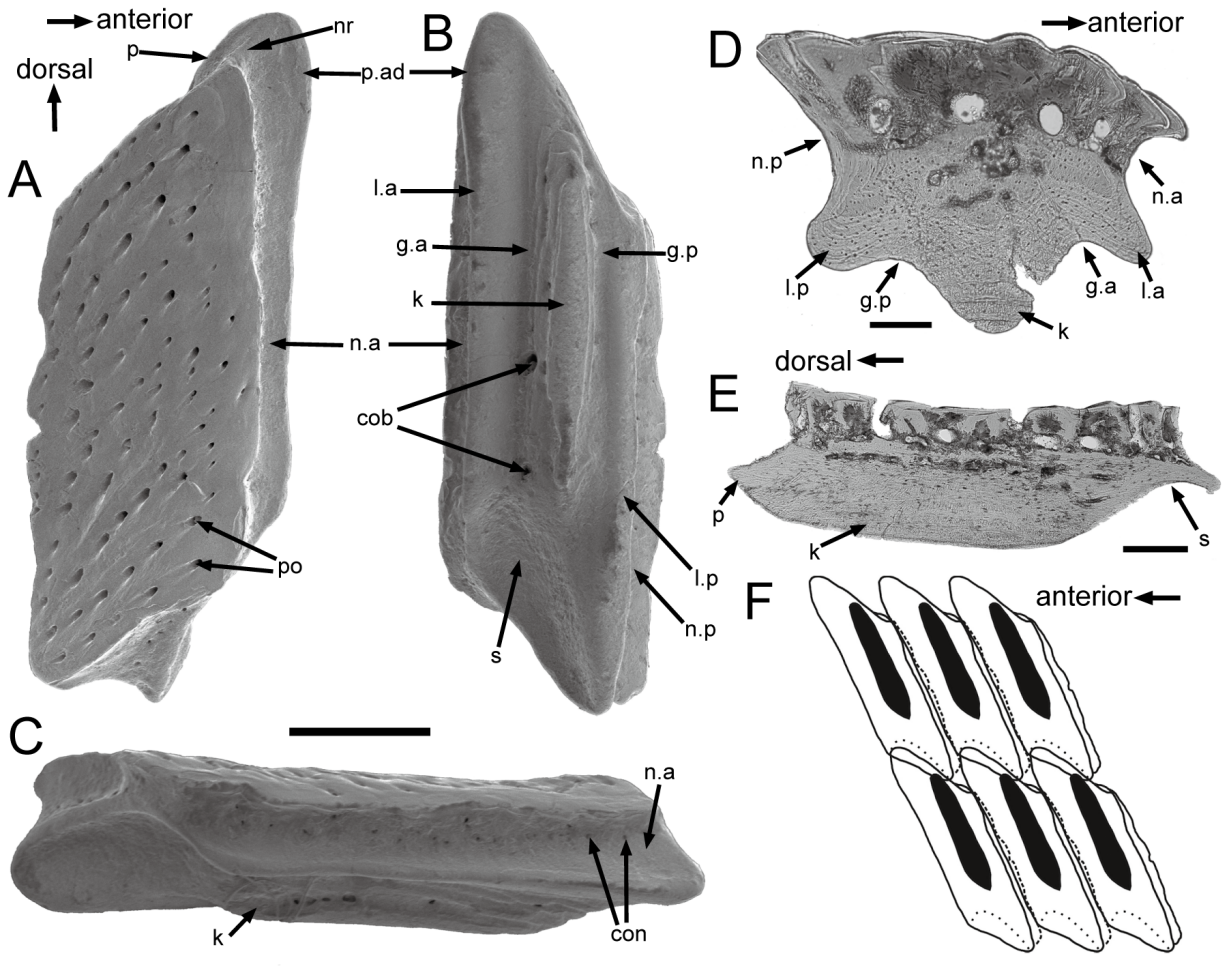
Gross anatomy of trunk scales (subtype 1) of *Psarolepis romeri* in surface view and ground sections. **A–C.** IVPP V17913.6 in crown view (A), basal views (B) and antero-lateral view (C); scale bar = 0.5 mm. **D.** IVPP V17757.16, light microscope photo, antero-posterior vertical ground section showing anatomical structures indicated in A–C; scale bar = 0.1 mm. **E.** IVPP V17757.17, light microscope photo, dorso-ventral vertical ground section cutting through the keel; scale bar = 0.1 mm. **F.** Reconstruction of the anterior squamation in basal view, showing the peg-and-socket structure *in situ*. cob, canal opening on the base; con, canal opening on the neck; g.a, anterior groove of the base; g.p, posterior groove of the base; k, keel; l.a, anterior ledge; l.p, posterior ledge; n, neck; nr, neck ridge; n.a, anterior neck; n.p, posterior neck; p, peg; po, pore opening on the crown; s, socket.

**Figure 4:**
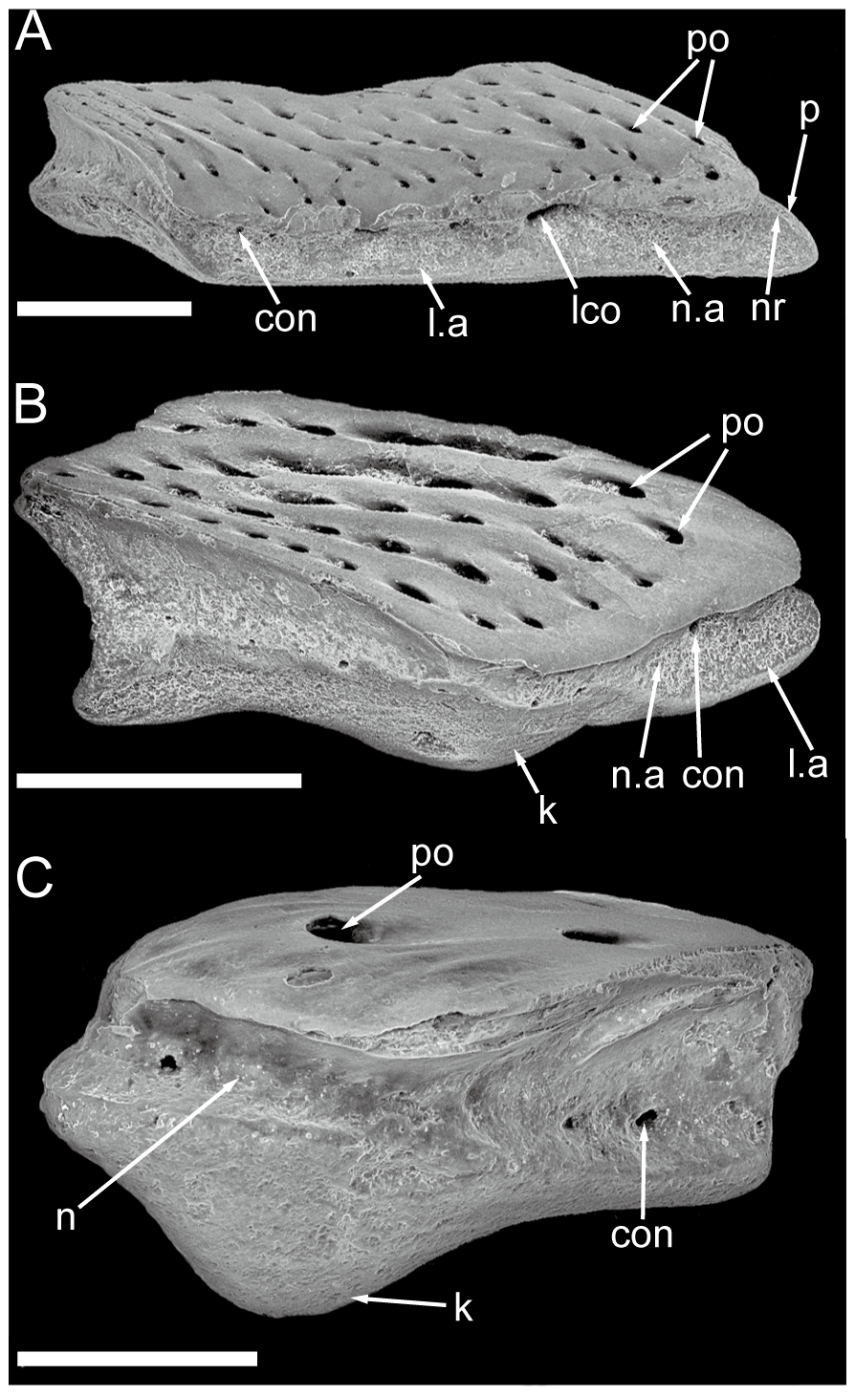
Selected scales displaying the neck structures. **A.** Antero-lateral view of IVPP V17913. 13, a subtype 1 scale with lateral-line canal, showing the lateral-line canal penetrating the neck in antero-posterior direction; **B.** Antero-lateral view of IVPP V17913.8, subtype 3; **C.** Lateral view of IVPP V17913.12, subtype 7. scale bar = 0.5 mm. con, canal opening on the neck; k, keel; l.a, anterior ledge; lco, lateral-line canal opening on the neck; n, neck; nr, neck ridge; n.a, anterior neck; p, peg; po, pore opening on the crown.

**Figure 5:**
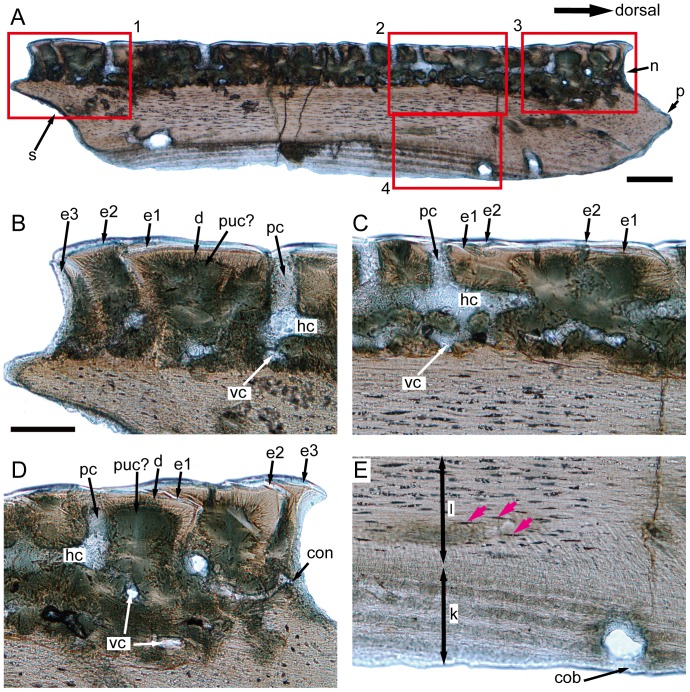
IVPP V17757.19, subtype 1, light microscope photos of a dorso-ventral vertical ground section. **A.** Full view of the ground section, the red insets are detailed in higher magnification in B–E, scale bar = 200 µm. **B.** The close-up of inset 1 in A, showing the odontode overlap pattern in the most ventral part of the crown, note that the pulp cavity is recrystallized and can only be recognizable according to the radiating dentine tubules; scale bar = 100 µm. **C.** The close-up of inset 2 in A, showing the odontode overlap pattern in the middle region of the crown, note the well-developed pore-canal system and the vascular canals connecting with the horizontal canals, the same scale bar as in B; **D.** The close-up of inset 3 in A, showing the odontode overlap pattern in the most dorsal part of the crown, the same scale bar as in B; **E.** The close-up of inset 4 in A, showing the boundary between the keel and the ledge, marked by the end of Sharpey's fibers, pink arrows indicate the osteocyte lacunae, note that only the keel shows the plywood-like pattern, the same scale bar as in B. cob, canal opening on the base; con, canal opening on the neck; d, dentine; e1–e3, enamel layers of first to third generations of odontodes; hc, horizontal canal; k, keel; l, ledge; n, neck; p, peg; pc, pore cavity; puc?, probable recrystallized pulp cavity; s, socket; vc, vascular canal.

All scales are thick, with a conspicuous neck separating the crown and the base (Figures 3 and 4). The neck is penetrated by small openings (con., Figures 3C and 4A). Ground sections show that these openings are connected with the vascular canal system inside the scale (Figure 5D). Antero-dorsally, the neck bears a septum-like ridge (nr, Figures 3A and 4A). In crown view, the crown almost shelters the base except the articulation portions (Figure 2). The rhomboid crown surface is ornamented with abundant pores, whose diameters range from 10 to 50 microns (Figure 2). All pores have higher anterior than posterior margins, forming a posteriorly-facing slope for each pore. Consequently, the large and closely spaced pores produce a slightly uneven surface. The pores have a fairly regular distribution, and are arranged into lines that generally extend parallel to the upper and lower margins of the crown. Anteriorly to the crown, a narrow strip is usually devoid of pores or has few small pores (Figures 2, 3A), and is curved downwards as shown in antero-posterior vertical ground section (Figure 3D). This curved strip is probably overlapped by the posterior extension of the crown of the adjacent scale.

**Figure 6:**
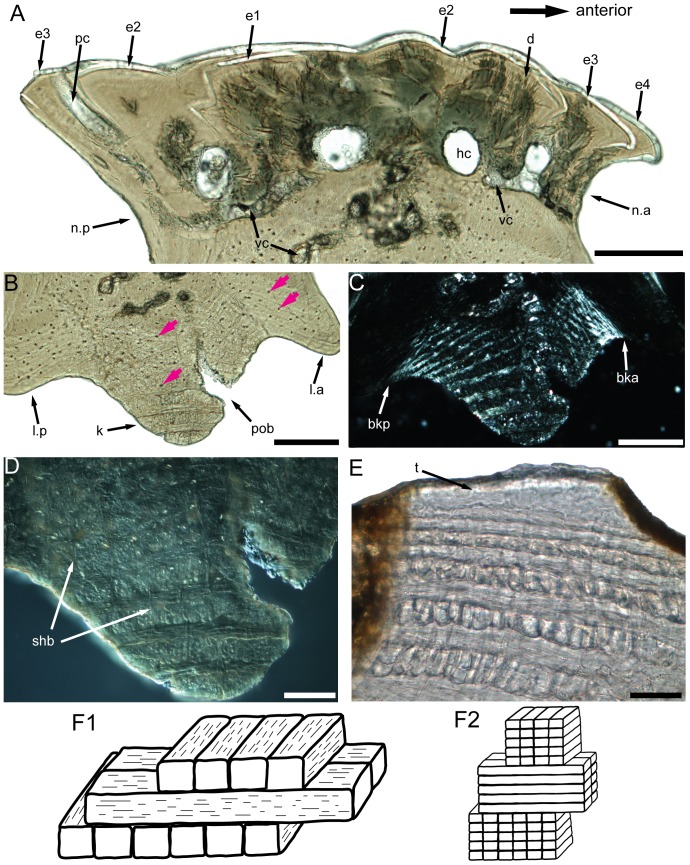
Comparison of the plywood-like tissues in *Psarolepis*, osteostracans and galeaspids. **A–D.** IVPP V17757.16, subtype 1, antero-posterior vertical ground section, showing light microscope photo of the crown (A) and the base (B), polarized light microscope photo of the base (C) and Nomarski interference light microscope photo of the keel (D), pink arrows indicating the osteocyte lacunae; scale bar = 100 µm in A–C, scale bar = 40 µm in D. **E.** IVPP V18540, vertical ground section through the dermal fragment of polybranchiaspid indet., showing the similar laminated pattern in galeaspidin but with less fiber layers in each ply; scale bar = 40 µm. **F.** Vertical ground section through the dermoskeleton of *Tremataspis mammilata* after Wang et al. [59], showing only one thick fiber-bundle in each ply; scale bar = 40 µm. **G.** Schematic models to compare the lamellated structures in osteostracans (G1) and keel of *Psarolepis* scales (G2), G1 is modified from Gross [13], note that the Sharpey's fibers are not incorporated; galeaspids have less fibril layers than *Psarolepis* in each ply, but more than osteostracans. bka, boundary between keel and anterior ledge; bkp, boundary between keel and posterior ledge; d, dentine; e1–e4, enamel layers of first to fourth generations of odontodes; hc, horizontal canal; k, keel; l.a, anterior ledge; l.p, posterior ledge; p, peg; pc, pore cavity; shb, Sharpey's fibers; t, tubercle on the top of galeaspid dermal skeleton; vc, vascular canal.

*Subtype 1* (Figures 2A, 3A–C, 4A, 5, 6A–D). The scales have a depth:length ratio of more than 1.5, comparable to that of the area A scales in the early actinopterygian *Moythomasia* ([34]: fig. 4A). 4 or 5 ridges are visible along the dorsal edge of the crown. The thick base bears a long protruding keel (k, Figure 3B) that is sandwiched in the anterior and posterior ledges (l.a, l.p, Figure 3B), thus forming two grooves in between (g.a, g.p, Figure 3B). Several openings are present on the keel and grooves. Ventrally, the base has a depressed area (s, Figure 3B), which corresponds to the antero-dorsal process of the base (p, Figure 3B) in shape. A reconstruction based on the outline of subtype 1 (Figure 3F) indicates that the ventral depressed area accommodates the antero-dorsal process of the base, thus forming a peg-and-socket articulation that is common in early osteichthyans [37]. A small process (p.ad, Figure 3A, B) protrudes anteriorly close to the dorsal end of the anterior ledge (Figure 3F).

A lateral-line scale, probably also from the area A of the body based on its depth:length ratio, shows that the lateral-line canal penetrates the neck. The lateral-line canal opening is much larger than other canal openings on the neck (Figure 4A). The lateral-line canal running through rather than between the scales has been considered as apomorphic for osteichthyans [37].

The scales assigned to subtype 1 most likely come from the most anterior flank of the body ([33]: Region A). In the articulated specimen of *Guiyu*, a close relative of *Psarolepis*, the anterior flank scales also have a depth:length ratio larger than 1.5 [11].

**Figure 7:**
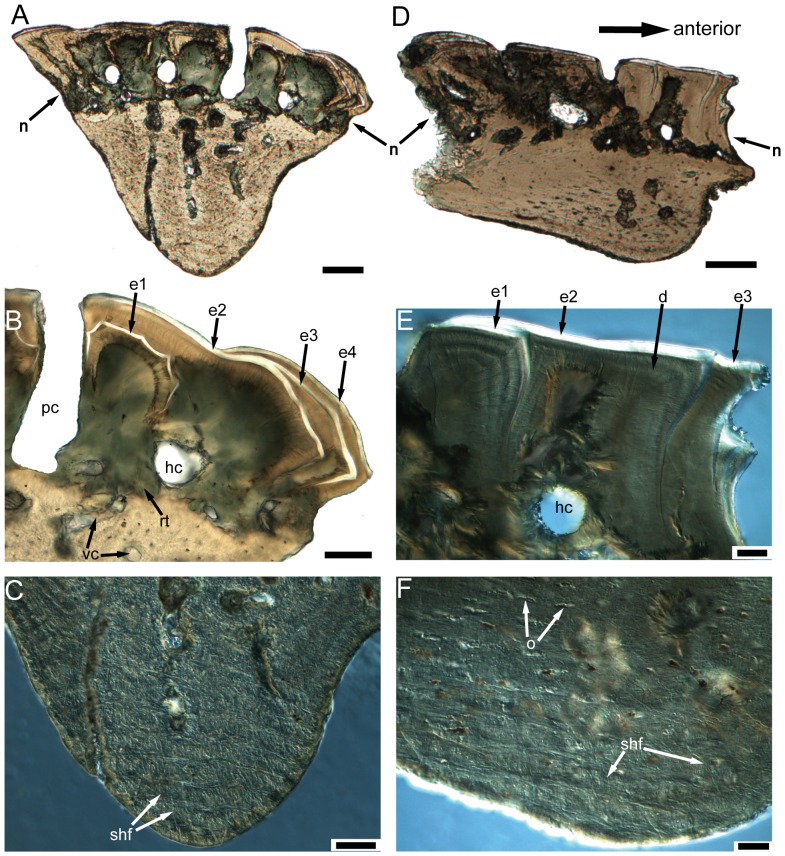
Scales (subtypes 2 and 4) of *Psarolepis romeri*. **A–C.** IVPP V17757.26, subtype 4, vertical ground section; A. Light microscope photo, full view; scale bar = 100 µm. B. Light microscope photo, close-up of A in the crown showing the overlap pattern of enamel layers; scale bar = 50 µm. C. Nomarski interference light microscope photo, close-up of A in the keel showing the plywood-like structure and Sharpey's fibers; scale bar = 50 µm. **D–F.** IVPP V17757.27, subtype 2, antero-posterior vertical ground section; D. Light microscope photo, full view; scale bar = 100 µm. E. Nomarski interference light microscope photo, close-up of D in the crown showing the overlap pattern of enamel layers; scale bar = 20 µm. F. Nomarski interference light microscope photo, close-up of D in the keel showing the plywood-like structure and Sharpey's fibers; scale bar = 20 µm. d, dentine; e1–e4, enamel layers of first to fourth generations of odontodes; hc, horizontal canal; o, osteocyte lacuna; pc, pore cavity; rt, recrystallized tissue; shf, Sharpey's fibers; vc, vascular canal.

*Subtype 2* (Figure 2B and 7D–F). The scales have a depth:length ratio of about 1.0, comparable to that of the area B scales in *Moythomasia* ([34]: fig. 4B). The number of lines of pores is less than that in subtype 1. The dorsal margin of the crown bears 7–8 ridges. The peg-and-socket articulation is less developed than that of subtype 1, and the antero-dorsal process is faint. The crown almost shelters the base, leaving a small corner of the base exposed in crown view (Figure 2B). Corresponding to the decrease in depth, the keel is also much shorter than that of subtype 1. The scales probably come from the middle flank of the body ([33]: Region B or C).

**Figure 8:**
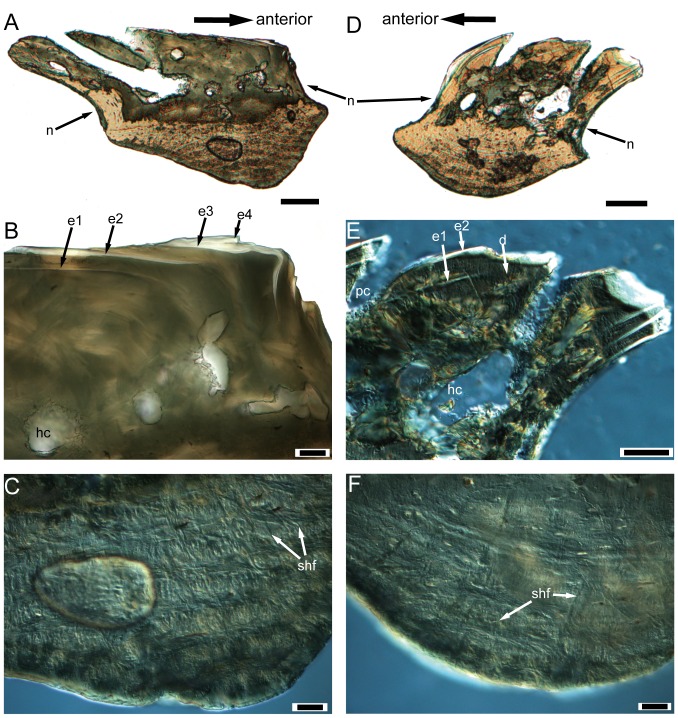
Scales (subtypes 3 and 7) of *Psarolepis romeri*. **A–C.** IVPP V17757.4, subtype 3, antero-posterior vertical ground section; A. Light microscope photo, full view; scale bar = 100 µm. B. Light microscope photo, close-up of A in the crown showing the overlap pattern of enamel layers; scale bar = 20 µm. C. Nomarski interference light microscope photo, close-up of A in the keel showing the plywood-like structure and Sharpey's fibers, note an air bulb on the central left; scale bar = 20 µm. **D–F.** IVPP V17757.28, subtype 7, antero-posterior vertical ground section; D. light microscope photo, full view; scale bar = 100 µm. E. Nomarski interference light microscope photo, close-up of D in the crown showing the overlap pattern of enamel layers; scale bar = 50 µm. F. Nomarski interference light microscope photo, close-up of D in the keel showing the plywood-like structure and Sharpey's fibers; scale bar = 20 µm. d, dentine; e1–e2, enamel layers of first to second generations of odontodes; hc, horizontal canal; pc, pore cavity; shf, Sharpey's fibers.

*Subtype 3* (Figures 2C, 4B, 8A–C and 9B). The scales have a depth:length ratio of about 0.5, comparable to that of the area D scales in *Moythomasia* ([34]: fig. 4E, F). The crown is usually longer than the base posteriorly, a feature more conspicuously shown in antero-posterior vertical ground section (Figure 8A). The base is nearly invisible in crown view (Figure 2C). Sometimes the scale is so low that the keel becomes a ball-like structure. The scales probably come from the posterior flank of the body ([33]: Region C or D).

In *Moythomasia*, the scales from the posterior trunk have a short, rounded keel, and a less-developed peg-and-socket structure than those from the anterior trunk [34]. Accordingly, the assignment of the subtypes 1–3 in an antero-posterior direction is in accordance with the pattern seen in *Moythomasia*. Subtypes 1, 2 and 3 are the most abundant among the referred scales, and more than 60 scales of these subtypes are used to make ground sections in this work. This abundance is consistent with their assignment to the trunk of the body.

*Subtype 4* (Figures 2D and 7A–C). The scales are symmetrical and elongated. The two anterior margins bear 5–6 ridges on each. The base is almost identical to the crown in size, and lacks any groove or ledge. This subtype has the same shape as the ‘pseudofulcral’ scales of *Andreolepis* ([38]: pl. 2), and might represent fulcral scales from the leading edge of the caudal fin.

*Subtype 5* (Figure 2E). The crown is elongated and extends beyond the base posteriorly, and the depth:length ratio (about 0.4) is even smaller than that of subtype 3. The peg, the socket and the anterior ledge are much broader than those in the subtypes 1–3. Because of the anterior extension of the base (anterior ledge) and the broadened peg, the ridge connecting the antero-dorsal corners of the crown and the base is also elongated. The keel is nearly ellipsoid or bulb-like. The scales, resembling the area F scales of *Moythomasia* ([34]: fig. 4G, H) in gross morphology, possibly come from the middle ventral flank of the body ([33]: Region F).

*Subtype 6* (Figure 2F). The crown resembles an irregular trapezoid. The anterior neck is less concave than in other subtypes. The base has a dorsal process (i.e., peg) and a short ventral extension that is not sheltered by the crown in crown view (Figure 2F). The scales, resembling the area H scales of *Moythomasia* ([34]: fig. 4K) in gross morphlogy, may come from the posterior ventral flank of the body close to the anal fin ([33]: Region H).

*Subtype 7* (Figures 2G, 4C and 8D–F). The scales are symmetrical like subtype 4, but less elongated. The crown is tiny (about 0.5 mm in mid-length) and bears few surface pores. About 2 ridges are visible along each anterior edge of the crown. The base bears a bulb-like keel (Figure 4C), but lacks the peg-and-socket structure. This subtype exhibits the general morphology of acanthodian scales [39] with its roundish outline, distinct neck and small size, however its histological composition (Figure 8D–F) differs from that of acanthodian scales. As no comparable scales are known in other osteichthyans, we suspect that this subtype might represent ridge scales as subtype 5, or scales covering the leading edge of fin web.

### (c) Scale histology

Given the similar histological composition in all subtypes, the description herein is mainly based on ground sections of subtype 1 scales. Differences between other subtypes and subtype 1 will be mentioned when necessary. The histological terminology will follow Francillon-Vieillot et al. [40] and Sire et al. [28].

In general, the referred scales are composed of three layers from crown to base: an upper cosmine-like layer (comprising enamel and dentine with canal system), a middle vascular bone layer, and a basal lamellated bone layer.

**Figure 10:**
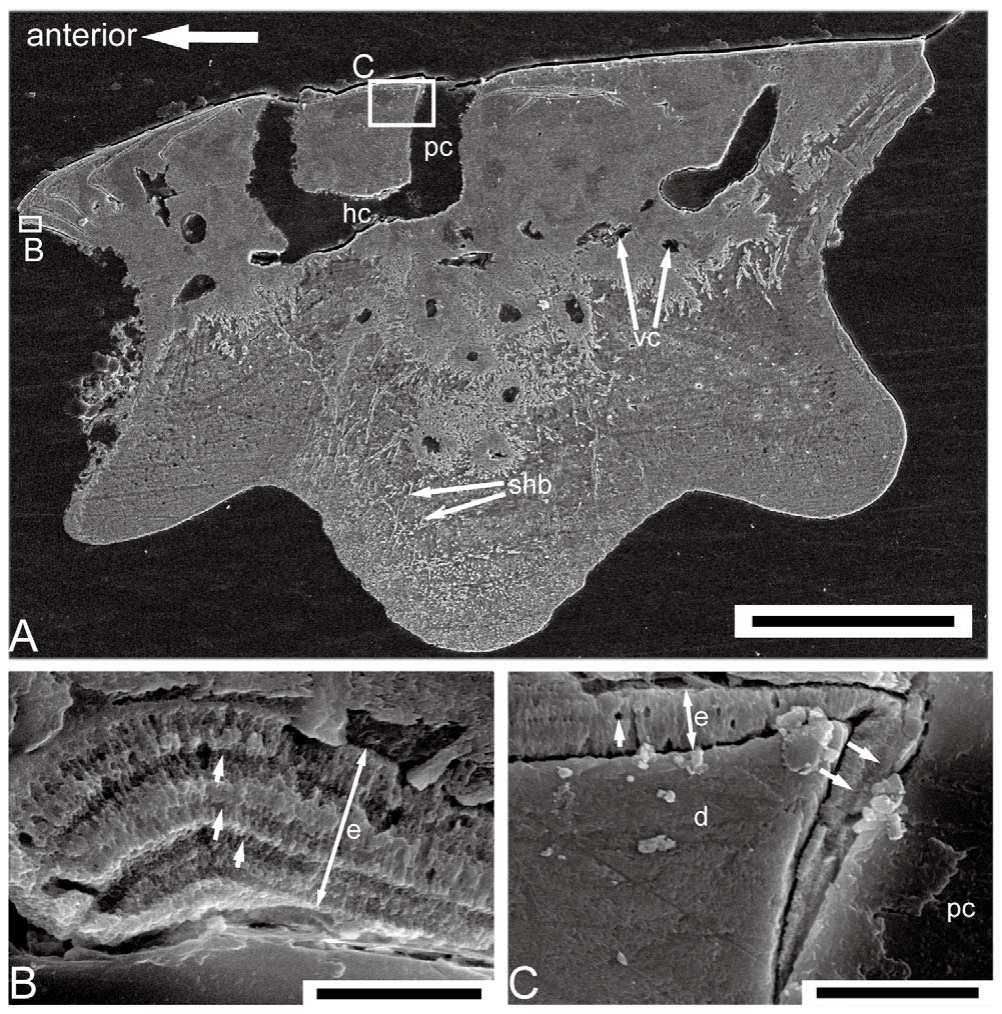
SEM picture of the etched ground section made from IVPP V17758, a subtype 1 scale. **A.** The full view of an etched ground section in antero-posterior direction; scale bar = 200 µm. **B.** The close-up of (A) showing the enamel crystallites and growth lines at the anterior margin of the scale, arrows marking the growth lines; scale bar = 5 µm. **C.** The close-up of (A) showing that the enamel dippers into a pore cavity; scale bar = 10 µm. d, dentine; e, enamel; hc, horizontal canal; pc, pore cavity; shb, Sharpey's fibers.

Most superficially in the cosmine-like layer is a hypermineralized layer, which is highly birefringent in transmitted light (Figures 5B, D and 6A). SEM study of the etched surface indicates that this layer consists of pseudoprismatic crystallites arranged in several layers that are separated by incremental lines (Figure 10B, C). The incremental lines, clear-cut boundary with dentine, and the pattern of pseudoprismatic crystallites suggest that this tissue represents the true enamel or monotypic enamel as discussed in Smith [41]. Fine tubules with branches permeate hard tissues under the enamel layer, and growth-lines are present in this layer (Figures 5D and 6A). These features are typical for dentine or orthodentine found in dermal skeleton of other early vertebrates [28], [42]. Dentine has been recrystallized in many parts (green under transmitted light), presumably indicating original locations of different cavities and canals (Figures 5B–D and 6A). Unlike the typical cosmine in crown sarcopterygians such as porolepiforms and lungfishes where only a single generation of enamel and odontodes are present [13], [27], [43], the cosmine-like tissue in referred scales is composed of multiple generations of enamel and odontodes, a condition that is similar to the dermal skeleton of *Meemannia*, *Styloichthys*, *Psarolepis* and primitive actinopterygians. This arrangement is considered primitive relative to cosmine found in some early crown sarcopterygians. [20], [21]. Enamel of younger generations of odontodes extends both superpositionally and areally relative to the enamel of older generations (Figures 5A–D, 6A, 7A, D, and 8A, D). The areal growth of odontodes is seen in four marginal regions (dorsal, ventral, anterior and posterior) of the scale, with the younger enamel layer extending to partially cover the surface of the adjacent older enamel layer (Figures 5B, D, 6A, 7B, E and 8B, E). Thus the scale surface is contributed to by enamel layers of different odontode generations, but the boundary between two enamel layers is difficult to determine. This growth pattern permits the scale to grow larger in area during the growth of the the body of the fish, while in more derived cosmine-bearing sarcopterygians a resorption-redeposition process was adopted to accommodate growth [44].

**Figure 11:**
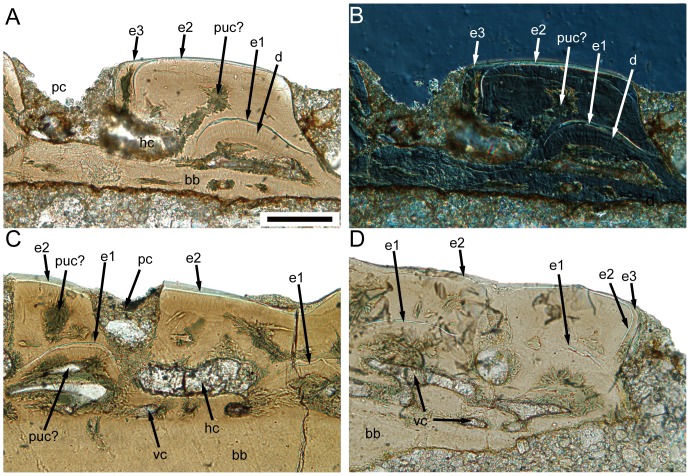
IVPP V17756, antero-posterior vertical ground sections of *Psarolepis* skull roof. **A.** Light microscope photo, showing three generations of odontodes on the bony tissue and the cylindrical pore cavity, note the vascular bone almost absent at this region, scale bar = 100 µm in all four figures; **B.** Nomarski interference light microscope photo of the same region in (A); **C.** Light microscope photo, showing two generations of odontodes and a thin vascularized layer below; **D.** Light microscope, showing three generations of odontodes with both superpositional growth and areal growth, the vascular bone well developed than in (A). bb, basal compact bone layer; d, dentine; e1–e3, enamel layers of first to third generations of odontodes; hc, horizontal canal; pc, pore cavity; puc?, probable recrystallized pulp cavity; vc, vascular canal.

A less-regular canal system, including a horizontal canal network and vertical pore cavities, is contained in the dental tissues. The pore cavities are slender and cylindrical in shape (Figures 5B–D, 7A, and 8A, D) rather than flask-shaped as in *Meemannia* [20] and more derived sarcopterygians [13], [27]. Such a cylindrical shape is similar to the condition in the dermal skull skeleton of *Psarolepis* as described below (Figure 11). Without horizontal ground sections being made, Zhu et al. [20] reconstructed the pore-canal system in the cranial dermal skeleton of *Meemannia* following the condition in *Porolepis* and *Osteolepis*, where each pore cavity sends out 4 to 5 basal branches ([13]: Maschencanals) to connect with the adjacent pore cavities. Two horizontal ground sections made from the referred scales show that the horizontal canal system is not as regular as in *Porolepis* or *Osteolepis*. The precise pattern (i.e., the number of adjacent pore cavities connecting with any given pore cavity) cannot be discerned, because the ground section levels are too deep into the bony tissues and not perfectly parallel with the horizontal canal system (Figure 9). Nevertheless, the horizontal canals do form a web-like network as shown by the connection of the dorsoventrally and anteroposteriorly oriented horizontal canals (Figure 9).

A bone layer under the canal network is penetrated by thin vascular canals, which connect with the overlying horizontal canals (Figures 5B, C and 6A). Compared with rhipidistian sarcopterygians, this vascular bone layer is much less developed. Due to the recrystalization around the canals, it is tentative to say that the horizontal canal system has a direct connection to the pulp cavities. However, further examination of the ground sections made from the dermal skull of *Meemannia* shows that the horizontal canals and pore cavities, like the lower vascular canals, do have a direct connection with pulp cavities in some cases ([21]: figures 5B and 6A). This is another major difference between the cosmine-like complex in *Meemannia* and the cosmine in rhipidistian sarcopterygians, where the pore-canal system does not connect with the pulp cavities directly [13]. Meanwhile, it is clear that the lower vascular canal system also contributes to the pulp cavities (Figure 5B–D). It is likely that the regular pattern of the pore-canal system separated from the pulp canals in crown sarcopterygians was attained in a stepwise way from the less regular pattern seen in *Psarolepis* and *Meemannia*.

Under the vascular bone layer lies the base, which consists of one keel flanked by two ledges (Figure 3D). Histologically the keel and two ledges are marked by two sharp lines under polarized light, and the two boundary lines (g.a, g.p, Figure 6C) usually lie at the deepest points of the two grooves on the base. For those scales without a distinct ledge region, the base is almost fully occupied by the keel (Figures 7A and 8A). Several differences are observed between the two types of tissues. While the tissue of the keel exhibits a plywood-like structure (Figures 5E and 6B–D), the two ledges show no such structure but only parallel fibers (Figures 5E and 6B). In the keel, each ply or lamella (approximately 20 µm in thickness) consists of several layers of parallel fibrils that are orthogonal to the adjacent lamella. Another set of extrinsic fibers, more obvious under polarized light (Figure 6C), penetrates only the keel, while the two ledges are devoid of extrinsic fibers (Figures 5E and 6C). These fibers are most comparable to Sharpey's fibers that anchor scales to underlying dermis and adjacent scale rows in the extant actinopterygian *Polypterus* (e.g. [45]). Small lacunae are abundant in the two ledges, and they are interpreted as osteocyte lacunae because of their size and fine canaliculi radiating from them (Figure 5E), although canaliculi are not always discernible because of inadequate preservation. The osteocyte lacunae are spindle-shaped in dorso-ventral vertical ground sections (Figure 5E). Compared with the two ledges, the keel has fewer osteocyte lacunae, which usually appear in the upper part of the keel (Figures 5E and 6B).

### (d) Dermal skull histology of *Psarolepis*

Zhu et al. [20] briefly described the dermal skeletal histology of *Psarolepis* based on a transverse ground section of a parietal shield. In this work, longitudinal ground sections are made from another unprepared parietal shield (IVPP V17756) of *Psarolepis*, revealing new information.

As reported in Yu [2] and Zhu et al. [3], surface of the skull roof in *Psarolepis* is punctuated by large and closely spaced pore openings. Similar to *Meemannia* [21], the dermal skeleton can be subdivided into three layers: the upper cosmine-like layer (odontodes separated by pore openings, pore cavities and interconnecting horizontal canals in the lower part), the thin vascular bone layer in the middle and the basal compact bone layer.

The comparison shows that the general patterns of the upper cosmine-like layer (a canal network embedded in superimposed layers of odontodes and enamel) are similar in *Psarolepis* and *Meemannia*, although the superimposed layers occur less frequently in the former. Based on new ground sections, additional differences have been revealed. First, the pore cavity in *Psarolepis* is approximately cylindrical in shape with uniform diameter across the depth, in contrast to the flask-shaped pore cavity in *Meemannia* and crown sarcopterygians such as *Youngolepis* and *Diabolepis* [27]. Some ground sections even show an enlargement of pore cavity upwards, giving the pore cavity a funnel-like shape (Figure 11A, C). Second, the dermal skull of *Psarolepis* displays areal growth (Figure 11A, D) in addition to the superpositional growth as previously described [20]. There are usually 2–3 generations of odontodes in the cosmine-like layer, with the second generation of odontodes superimposed on the first generation (Figure 11). However, when there is a third generation, the odontodes usually lie beside the second generation, showing an areal growth pattern (Figure 11A, D) that is not observed in *Meemannia* or *Styloichthys* [20], [21]. This growth pattern is similar to that in the marginal regions of the scales.

The vascular bone below the horizontal canal system is poorly developed in *Psarolepis* (Figure 11C, D). Occasionally, the compact bone layer lies directly under the cosmine-like layer (Figure 11A). Although initially described as the lamellar bone [20], the inner compact bone tissue lacks any plywood-like pattern that is characteristic for the lamellar bone [28], [40]. This compact bone layer is more comparable to the bone tissues constructing the two ledges in the scales described above, and devoid of any extrinsic fibers.

## Discussion

### (a) Plywood-like tissue constructing the scale keel

The scale keel in *Psarolepis* is composed of a type of plywood-like tissues, with about 8–13 collagen plies [40], [46], [47], [48]. Each ply consists of several layers of fiber-bundles that are parallel to each other but orthogonal to the fiber-bundles of the adjacent ply (Figure 6D, F2). Orthogonal to these intrinsic fiber-bundles is a set of extrinsic thick fibers penetrating multiple plies (Figures 5E, 6C, D, 7C, F, and 8C, F). When viewed under polarized light, the keel exhibits alternating stripes of black and white (Figure 6C). This structure conforms well to the definition of the lamellar bone [40]. By comparison, the rest of the scale base including the flanking ledges always exhibits a homogenous pattern and does not show any plywood-like organization. A boundary between the keel and the rest of the base is usually evident when examined under polarized light (Figure 6C). By definition, the collagen tissue in the rest of the base is a type of pseudo-lamellar bone or parallel-fibered bone [40].

It needs to be pointed out that in this paper we follow the terminology in Francillon-Vieillot et al. [40] and use the term “lamellar bone” only to refer to the lamellated bone with a plywood-like structure. Meanwhile, the term ‘isopedine’ is adopted to describe a subtype of lamellar bone (either cellular or cellular) with an orthogonal plywood-like structure [contra Meunier [47] who employed isopedine to describe elasmodine in teleosts]. In this paper, isopedine is interchangeable with lamellar bone as no twisted plywood-like structure is involved.

Comparison with the rhombic scales of other early osteichthyans shows that the histological organization in the scale base of *Psarolepis* is unique. In the trunk scale of *Ligulalepis*, the bony base is constructed by homogenous cellular lamellated bone, with Sharpey's fibers restricted in the keel [49]. The scale base in *Andreolepis* is composed of homogenous cellular bone without any plywood-like organization and with Sharpey's fibers restricted in the keel [50]. In *Moythomasia* and *Mimipiscis*, the scale base was also described to be composed of homogenous cellular lamellated bone penetrated partially by Sharpey's fibers, but the microstructure of the lamellated bone was not illustrated [51], [52]. Traditionally, many authors employed the term ‘lamellar bone’ to describe the lamellated bone (e.g. [20], [49], [51], [52], [53]). The ‘lamellar bone’ in these works does not necessarily show a plywood-like pattern and might be the pseudo-lamellar bone sensu Francillon-Vieillot et al. [40]. For example, the ‘lamellar bone’ in the dermal skull of *Meemannia* and *Psarolepis* [20] can be referred to the pseudo-lamellar bone because of its homogenous nature under polarized light. In *Polypterus*, the bony base of the scale has been described as constructed by homogenous pseudo-lamellar bone, and Sharpey's fibers are also restricted to the keel [45], [54], [55].

The histological organization of the scale base in the porolepiform sarcopterygian *Heimenia* [43] is also different from that in *Psarolepis*. The scale keel ([43]: ‘internal bone layer’) in *Heimenia* does not show any plywood-like structure. However, the rest of the scale base ([43]: ‘basal layer’) is composed of lamellar bone showing a plywood-like structure. In addition, both the keel and the rest of the base are penetrated by Sharpey's fibers. Other early sarcopterygians such as *Porolepis* and *Osteolepis* also have typical cosmoid scales, whose base (excluding the keel) is composed of a thick layer of isopedine [13]. The keel was described as constructed by spongy bone in Gross [13].

To summarize, no plywood-like tissue has been found in the keel of the scale among osteichthyans except *Psarolepis*. However, the plywood-like tissue is present in the base of non-rhombic scales referred to some acanthodians [56], [57] and putative early chondrichthyans ([58]), suggesting the possibility that the keel microstructure in *Psarolepis* may represent a retained primitive feature for osteichthyans. Like the scale keel of *Psarolepis*, the base of non-rhombic scales of some acanthodians and putative chondrichthyans is also constructed by intrinsic isopedine plus extrinsic Sharpey's fibers, although the thickness and pattern of the Sharpey's fibers show some variations in different taxa [56], [57], [58]. The keel of rhombic scales is the most interior part and functions as a structure to connect the scales with the subdermis, usually indicated by the presence of Sharpey's fibers (e.g. [45]). This is also the case for the bony base of acanthodian scales [56], [57] and putative early chondrichthyan scales [58], implying that this bony base might be homologous to the keel of rhombic scales.

It is noteworthy that the microstructure of the plywood-like tissue in *Psarolepis* resembles that of galeaspidin, an enigmatic tissue only known from galeaspids [59], an early jawless vertebrate group endemic to China and Vietnam [9], [60], [61]. The dermal skeleton of galeaspids is composed of two types of tissues, the galeaspidin in the inner thick layer and the microspherulitic acellular bone in the outer capping layer [59] (Figure 6E). The intrinsic collagen fibrils of galeaspidin form an orthogonal plywood-like tissue that is similar to isopedine as in some osteostracans [13], [62], [63]. However, for each ply, galeaspidin has several thinner layers of parallel fibrils (as in the plywood-like tissue of *Psarolepis*) while the osteostracan isopedine has only one layer of thick fibrils (Figure 6F1, F2). In addition, galeaspidin has other thick extrinsic fibers (Sharpey's fibers) penetrating the entire depth. Although galeaspidin has been explained as metaplastic ossification of the stratum compactum of dermis [28], the tissue composition of galeaspidin prompts comparison to the plywood-like tissue in *Psarolepis*, which equally suggests its nature as a type of lamellar bone [59].

### (b) Evolution of rhombic scales in early osteichthyans

The rhombic scale, a diagnostic feature of osteichthyans [37], can be defined by its rhomboid shape with long diagonal axis, and a long keel flanked by two grooves in the base. Usually, the peg-and-socket articulation (either broad or narrow) exists between adjoining rhombic scales. The rhombic scales are also known from some placoderms and jawless fishes such as osteostracans and anaspids, however, their base structures and histology show no similarity to those in osteichthyans [9].

Conventionally, the rhombic scales can be classified into two groups: ganoid and cosmoid scales [28], [64]. Schultze [64] thoroughly discussed early evolution of rhombic scales in osteichthyans, and proposed a scenario that ganoid and cosmoid scales evolved from a *Lophosteus*-like scale morphotype. Based on this scenario, the referred scales (subtypes 1–7) can be identified as cosmoid-like scales because of the presence of canal system and underlying vascular bone layer. However, these scales also show areal or lateral addition of new enamel layers and odontodes, a feature that is characteristic of ganoid scales and absent in any known cosmoid scales. The growth pattern of enamel is comparable to that in the scales of *Andreolepis* [50], *Moythomasia* [52] and other primitive actinopterygians, where the young marginal enamel layer only partially overlaps the older layer. Unlike the geologically younger actinopterygian taxa *Palaeoniscum* and *Lepisosteus* [65], the enamel in *Psarolepis* scales never grows in an onion-like pattern. In addition, the canal system and the vascular bone layer are less developed than in cosmoid scales of more derived sarcopterygians (e.g. *Porolepis* and *Osteolepis*), where one pore cavity sends out 3–5 basal branches to connect with adjacent pore cavities, forming a regular grid-like horizontal canal network [13]. By comparison, the horizontal canals in the referred scales constitute an irregular web, dominated by antero-posteriorly oriented canals (Figure 9). Under the prevailing phylogenetic framework [7], [11], the less regular horizontal canal system in *Psarolepis* scales represents a plesiomorphic state of sarcopterygians. The vascular bone layer, as in the dermal skull of *Meemannia* and *Psarolepis* [20], is much thinner than in *Porolepis* and *Osteolepis*. To sum up, the scales of *Psarolepis* combine the characters of ganoid and cosmoid scales, and might provide a new model for discussing the origin of ganoid and cosmoid scales.

**Figure 12:**
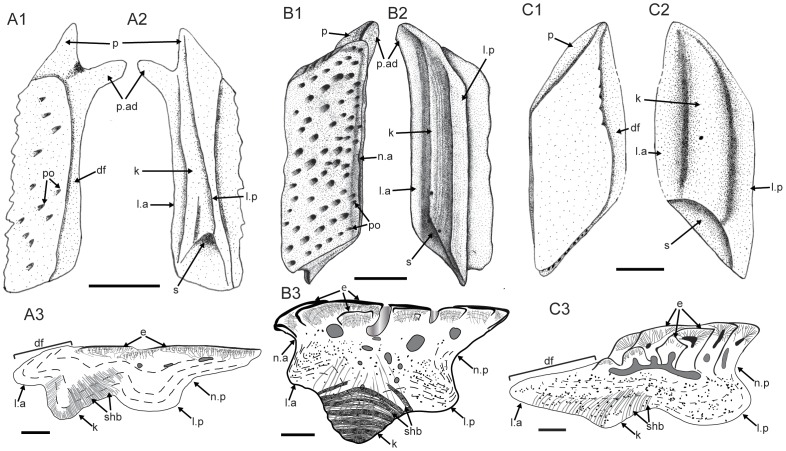
Comparison of rhombic trunk scales of three early bony fishes, in illustrative drawings. **A.** *Ligulalepis toombsi*, modified from Burrow [35]; **B.** *Psarolepis romeri*; **C.** *Andreolepis hedei*, adopted from Gross [50]. The second keel (k′) in Schultze [49] is explained as the posterior ledge that has the same tissue composition as the anterior ledge, but is different from the keel. Scale bar = 0.5 mm in A1–A2, B1–B2 and C1–C2; scale bar = 0.1 mm in A3, B3 and C3. df, depressed field; e, enamel; k, keel; l.a, anterior ledge; l.p, posterior ledge; n.a, anterior neck; n.p, posterior neck; po, pore opening; shb, Sharpey's fibers.

It is noteworthy that the referred scales (subtypes 1–7) bear a distinct neck separating the crown and base, and lack the depressed field as seen in other rhombic scales (df, Figure 12A1, B1, C1). Comparison with lateral-line scales of *Moythomasia* ([52]: fig. 142) and *Mimipiscis* ([52]: fig. 141) indicates that the ventral part of the anterior neck or the dorsal surface of the anterior ledge corresponds topologically to the depressed field where the lateral-line canal enters the scale anteriorly. A depressed field of the scale is evident in *Ligulalepis* and *Andreolepis* (Figure 12A1, C1), although the scale thickness in these two forms is comparable to that in *Psarolepis*. Without a depressed field, the overlap pattern of adjacent scale rows in *Psarolepis* might differ from that in other early osteichthyans. If we follow the model in other osteichthyans [9], we might consider that the scale in the front overlaps the scale behind it on the dorsal surface of the anterior ledge, which is a topological equivalent of the depressed field in other osteichthyans. However, this overlap relationship is not functional as it will impede the posterior growth of the crown of the scale in front. Alternatively, we consider the scale in the front overlaps the scale behind it along the anterior, downward-curving belt of the crown, which functionally corresponds to the depressed field, but is not part of the bony base as in other rhombic scales.

The posterior ledge in the referred scales correspond*s* to the second keel (k′) identified by Schultze [49] on the scales of *Ligulalepis* (Figure 12A). In the referred scales, only subtype 4 exhibits a prominent anterior ledge that is not sheltered by the crown, thus forming a structure similar to the depressed field (Figure 2D). The lack of the depressed field in other types may be due to the growing odontode encircling the crown, thus making the neck concaved and invisible in crown view. Given the fact that the neck is widely present in non-osteichthyan jawed vertebrates (e.g. placoderms and acanthodians; [39], [66]), it is tempting to explain the distinctive neck in the scales of *Psarolepis*, like the dermal pelvic girdle in *Psarolepis* [32], as a retained primitive gnathostome feature. However, the prevailing hypotheses of relationships among early osteichthyans [11], [37], which resolve *Psarolepis* as a stem sarcopterygian, would favor the interpretation that the scale neck in *Psarolepis* is an apomorphic reversal to the plesiomorphic condition. This character discrepancy with the prevalent phylogenies calls attention to the alternative scenario, which places *Psarolepis* as a stem osteichthyan [3], [10], [32]. A more comprehensive phylogenetic analysis incorporating the scale characters revealed in this study is needed to test whether the neck in the scales of *Psarolepis* is a primitive retention or an apomorphic reversal.
